# The Moderation Effect of Processing Efficiency on the Relationship Between Visual Working Memory and Chinese Character Recognition

**DOI:** 10.3389/fpsyg.2020.01899

**Published:** 2020-08-05

**Authors:** Zhengye Xu, Li-Chih Wang, Duo Liu, Yimei Chen, Li Tao

**Affiliations:** ^1^Department of Special Education and Counselling, The Education University of Hong Kong, Tai Po, Hong Kong; ^2^Integrated Centre for Wellbeing, The Education University of Hong Kong, Tai Po, Hong Kong; ^3^Centre for Child and Family Science, The Education University of Hong Kong, Tai Po, Hong Kong; ^4^Shenzhen Tongle School, Shenzhen, China; ^5^Xuefu Middle School, Shenzhen, China

**Keywords:** processing speed, serial order, temporal sensitivity, visual processing, word recognition

## Abstract

To investigate the underlying mechanism of the relationship between visual working memory (VWM) and Chinese character recognition, and the moderation effect of processing efficiency on this relationship, 154 first-grade students were administered a battery of tasks for VWM, rapid temporal processing, and Chinese character reading. In the VWM task, the children were asked to remember the jumping routes of a frog and report these routes in reverse sequence. The longest span for which each participant could respond correctly at least four times out of six was the VWM index. In the task of temporal order judgement, the participants were asked to select which of two balls was presented first, with stimulus onset asynchronies varying from 8 to 492 ms according to an adaptive psychophysical procedure. Visual temporal order threshold (VTOT) was utilized as an indicator of processing efficiency. The participants were asked to read 100 characters aloud to measure their word-level reading abilities in Chinese character recognition. After controlling age, non-verbal intelligence, visual short-term memory, morphological awareness, and orthographic awareness, the results of a moderation effect analysis showed that (1) both VWM and visual VTOT predicted Chinese character reading, and (2) the moderation effect of VTOT on the VWM-reading link was significant (*p* = 0.001). The correlation between VWM and Chinese character reading was positive and significant when VTOTs were above average (i.e., smaller than 87.14 ms); however, the correlation was negative at relatively poor levels of VTOTs (i.e., larger than 231.44 ms).

## Introduction

Working memory (WM) is the ability to concurrently store and manipulate information that is necessary to perform mental tasks (Baddeley et al., 1986). WM capacity is generally measured by WM span tasks, in which participants are asked to maintain items to be recalled while they perform a demanding secondary task such as counting (Baddeley, 2003). Compared with the literature on phonological WM (Hansen and Bowey, 1994; Calvo, 1996; Chung et al., 2010), the literature on visual WM (VWM) and reading is limited, and the findings are controversial (e.g., Olson and Datta, 2002; Jeffries and Everatt, 2004; Smith-Spark and Fisk, 2007). VWM, as an acritical component of WM (Baddeley and Lieberman, 2017), is thought to be important for beginning readers’ word recognition, especially in languages with logographic writing systems, such as Chinese (Huang and Hanley, 1995; Wang et al., 2014, 2015).

The inconsistent findings regarding the association between VWM and reading might be due to individual differences in aspects related to WM and/or reading, such as age and reading experience (e.g., Gottardo et al., 1996; Berninger et al., 2010; Sullivan et al., 2015). The capacity of VWM can be improved with the increase of processing efficiency (Olson and Jiang, 2004; Chung et al., 2008; Brady et al., 2009; Wang et al., 2019). Children with the same VWM capacity, but different levels of processing efficiency, should utilize different cognitive resources on the same task (Barrouillet et al., 2004). In addition to influencing VWM performance, children’s processing efficiency might further impact higher-level cognitive processing that is related to VWM, such as reading. To address these issues, the present study investigated the association between VWM and Chinese character reading in first-grade Chinese students. With an index of visual processing efficiency (i.e., rapid temporal processing, the possible role of processing efficiency in this association was explored. The findings can extend our understanding of the underlying mechanism of the relationship between VWM and Chinese character recognition. In turn, understanding the mechanism could help us develop efficient means to improve children’s reading.

### WM and Chinese Character Recognition

Working memory consists of multicomponent memory systems. Phonological WM, i.e., the phonological loop, has a phonological store and an articulatory rehearsal system (Baddeley et al., 1998). VWM, also known as the visual-spatial sketchpad, is used for immediate maintaining and processing of visual non-verbal information, including visual features and spatial analysis (Peng et al., 2013). These two WM systems, together with the central executive system and a newly proposed episodic buffer, form a complete system and are effective in explaining reading and other cognitive processes (Baddeley, 2003). The effect of WM on reading is demonstrated by findings that children with reading disabilities show significant and marked decrements on WM tasks compared to typically developing children in alphabetic languages (e.g., Siegel and Ryan, 1989; Swanson et al., 1996; Swanson, 1999) and in Chinese (e.g., Luo et al., 2013; Xu et al., 2015). One explanation of how deficits of WM affect reading is that poor WM capacities impede a crucial reading process, i.e., maintaining recently retrieved knowledge and integrating it with the present inputs (Swanson and Beebe-Frankenberger, 2004).

The association between WM and word recognition has been investigated extensively, mainly by focusing on phonological WM (e.g., Gathercole and Baddeley, 1993; Baddeley, 2003; Chee et al., 2004; Leong et al., 2008) rather than VWM (Opitz et al., 2014; Peng et al., 2013). More importantly, findings about the association between VWM and reading are inconclusive in alphabetic languages. Some studies have documented deficits in VWM associated with poor reading performance (Olson and Datta, 2002; Smith-Spark and Fisk, 2007), but others failed to find any such association (Jeffries and Everatt, 2004; Kibby et al., 2004).

Studies of the impact of VWM on word recognition in typically developing Chinese children are scarce (e.g., So and Siegel, 1997). However, Chinese has unique features that might highlight the role of VWM in reading. Specifically, different from alphabetic languages, Chinese characters map onto phonology at the syllable level, which means that the usage of grapheme-phoneme correspondence rules is not possible in Chinese (Tan et al., 1996; Perfetti et al., 2005). That is, it is difficult for Chinese readers to rely on grapheme-phoneme conversion rules to transfer written words into a phonological code automatically. When processing characters, the sounds need to be retrieved through their links with corresponding visual forms in the mental lexicon (Shu et al., 2003; Ho et al., 2007).

**FIGURE 1:**
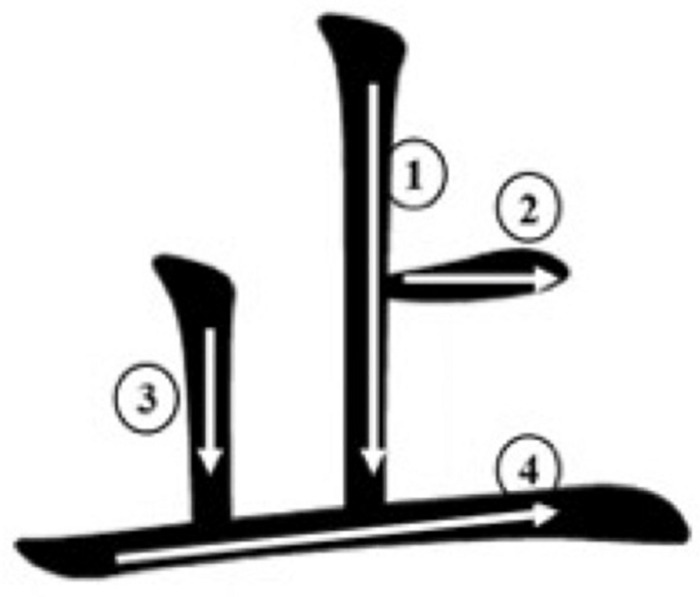
The stoke order of the Chinese character *止*/zhi3/ (stop).

In addition, the basic unit of Chinese, the character, is composed of strokes that are packed into a square configuration, processing a high, non-linear visual complexity. A simplified Chinese character has 10.3 strokes on average (Srihari et al., 2007). As a system for processing visual information, VWM may benefit the acquisition and recognition of the complex visual features of characters, which can further influence reading performance in Chinese (Tan et al., 1996; Siok et al., 2009). A mainly effective way for Chinese children to learn characters is repetitive writing (Tan et al., 2005). During this process, VWM should help children to maintain the current stimuli (e.g., strokes) and retrieve their stroke knowledge, and then follow the specific sequence to construct the form of a character. For example, when learning the character止/zhi3/ (stop), children need to process the form and stroke order at the same time to retrieve the knowledge of how to write the strokes, i.e., 丨一, and then to construct the character properly (see Figure 1). The contribution of VWM to t character acquisition should be reflected further as a relationship between VWM and character recognition.

As well, the literature demonstrates that the segments of forms (e. g., strokes and radicals) can be processed to facilitate character recognition (Li et al., 2000; Liu et al., 2010). Many characters look very similar but have totally different meanings, such as
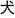
\da4\(big), 太\tai4\(too), and 犬\quan3\(dog). To reach reading, children need to process the visual features and, simultaneously, to distinguish them from others with similar visual forms in their mental lexicons, which is related to VWM (Just and Carpenter, 1992; Shu et al., 2006). Considering that both Chinese character acquisition and recognition require simultaneous processing and storage of these visual segments of characters, which are the core nature of WM (Baddeley et al., 1998; Gathercole and Baddeley, 1993), it is reasonable to postulate the association between them.

### Processing Efficiency in VWM and Chinese Character Recognition

It has been suggested that VWM is a limited-capacity system in which some resource is shared between processing and storage (Baddeley, 2003). Such a limited-capacity system leads to a phenomenon that the performance of VWM decreases when the concurrent memory load increases (Anderson et al., 1996; Baddeley, 2012). Nevertheless, it was suggested that the capacity of VWM can be influenced by processing efficiency (Brady et al., 2009). For instance, generally, an age-related increase occurs in the counting span task due to an enhanced efficiency to process sequences of numbers. Interestingly, this developmental increase of VWM capacity disappeared when participants were asked to count using unfamiliar terms, which reduced the processing efficiency in the counting task (e.g., Case et al., 1982; Bull and Scerif, 2001). On the other hand, some studies found that enhancing the processing efficiency of input (e.g., involving statistical structure and regularities) can lead to the improvement of VWM capacity (Olson and Jiang, 2004; Olson et al., 2005).

The time-based resource-sharing (TBRS) model (Barrouillet et al., 2004) assumed that, in tasks for VWM capacity, both processing and maintenance of information rely on the same limited cognitive resource (e.g., attention). High processing efficiency facilitates the VWM performance by assisting a rapid and incessant switching of cognitive resources from processing to maintenance. This rapid switching occurs during short pauses, which occurs when concurrent processing is running. Accordingly, compared with continuously occupying attention, the frequent pauses and switching can decrease demanding. Hence, the high processing efficiency improves VWM performance through lightening the cognitive load (Barrouillet et al., 2007). In turn, it is reasonable to assume that, if individuals have equal VWM capacities, these with higher processing efficiency can use fewer cognitive resources to process the same content.

It should be noted that, in addition, to processing visual features, other cognitive processing (e.g., processing of phonology) is required in Chinese character recognition. However, humans’ cognitive resources are limited (Paas et al., 2003); the more they are used for visual processing and maintenance, the less remains for the other cognitive processing. According to the TBRS model (Barrouillet et al., 2004), although children with different levels of processing efficiency can have the same VWM capacities, the cognitive resources that they use for the task of VWM are different. Of children with the same VWM capacity, these with high processing efficiency will use less resources than those with low processing efficiency to process and maintain visual features of characters. Thus, these children with high processing efficiency can have more cognitive resources available for cascade processing, such as activating the corresponding sounds of the visual forms (Baddeley, 2003). As a consequence, their performances in Chinese character recognition might be improved to larger extents (van Merrienboer and Sweller, 2005).

In brief, according to the TBRS model (Barrouillet et al., 2004), the current study assumed that children with the equal VWM capacity but different levels of processing efficiency could process visual features to similar extents, but that the cognitive resources that they use for processing these visual features would be different. That is, their available cognitive resources for other aspects of Chinese character recognition should be different (Barrouillet et al., 2007). With more remaining cognitive resources, the children with high processing efficiency could score better on Chinese character recognition tasks. In contrast, low processing efficiency might lead to a lack of resources for the cascade processing of character recognition. As a consequence, the association between VWM and Chinese character recognition could be strengthened in children with high processing efficiency, whereas it might be weakened in those with low processing efficiency.

### Present Study

In sum, the current study recruited children to complete tasks of visual temporal order judgement (TOJ), backward Corsi, and Chinese character reading for their visual processing efficiency, VWM, and Chinese character recognition, respectively. The visual TOJ task involves a paradigm for measuring visual rapid temporal processing. Visual temporal processing is a more fundamental cognitive ability that relates to processing brief components and rapid sequences of information, which has a facilitating effect on Chinese character recognition (e.g., Chung et al., 2008; Wang and Yang, 2018; Wang et al., 2019). The relationship between VWM and Chinese character recognition and the role of processing efficiency in the relationship were investigated.

Since there are close correlations between beginning readers’ basic cognitive abilities (e.g., WM and temporal processing) and their reading (Schneider and Pichora-Fuller, 2001; Cain et al., 2004), first grade could be a good starting point for exploring the relationships between VWM, processing efficiency and reading in Chinese. Considering their impacts on VWM and reading in children (Swanson et al., 2009), age and non-verbal intelligence were controlled. In addition, as the current study focused on the core component of VWM, i.e., the ability to process and maintain concurrently (Baddeley, 2003), visual short-term memory (VSTM) was measured. Hence, the role of VWM in character recognition was able to be investigated independently of the influence of VSTM (Cowan, 2017), which reflects storage of information in a short time (Engle et al., 1999). In addition, in order to further control the influence of children’s metalinguistic abilities on Chinese character recognition (Liu and McBride-Chang, 2010; Li et al., 2012), morphological awareness and orthographic awareness were taken into account as covariates.

## Materials and Methods

### Participants

A total of 154 Chinese children (90 boys and 64 girls) in Grade 1 (mean age = 7.17 years, SD = 0.35) participated in this study. All participants were native speakers of Chinese and were recruited from a primary school located in an urban community with a low-to-middle socioeconomic level, in Shenzhen, China. Written informed parental consent was obtained for the participants. The schools and parents reported no physical or mental problems in any of the children.

### Procedure

Each participant was asked to complete the VWM task, the visual TOJ task, the Chinese character reading task, and the control measures individually, in a quiet classroom. These tasks were administered by trained experimenters and required approximately 1 h to complete.

### Measures

#### Corsi Forward and Backward Tasks

A Forward Corsi task was adopted to test the participants’ VSTM, and a backward Corsi task was adopted to measure VWM (Ang and Lee, 2008). Both tasks were performed using an E-prime program. First, a picture with nine lily pads was presented, and then, a frog jumped across the lily pads to go somewhere. In the Corsi forward task, the participants were asked to remember and repeat the order by clicking the corresponding lily pads sequentially, while, in the backward task, they were asked to indicate the backward order for each trial. The forward task was started from one lily pad, whereas the backward task was started from two lily pads. The number of lily pads increased by one when a participant answered correctly four times for the six sequences; otherwise, the task was terminated. The highest number of lily pads that participants could remember (i.e., four correct responses out of six sequences) in the Corsi forward and backward tasks were the scores for VSTM and VWM, respectively. The maximum score for each task was nine.

#### Visual TOJ Task

A visual TOJ task that was used in a previous study (Steinbrink et al., 2014) was applied to measure children’s visual rapid temporal processing. The task utilized visual temporal order thresholds (VTOTs) as the indicator of visual temporal processing, which increased the efficiency and reliability of the measurement (Leek, 2001; Wittmann and Fink, 2004). The smaller the value of VTOT, the higher the performance in the visual TOJ task, that is, the higher the temporal sensitivity. An adaptive psychophysical procedure was used to measure individual thresholds in this task. The distance between the participant and screen was 30 cm. A scenario with a seal was presented in the middle of the scene at first and, after a delay of 500 ms, one of two colored balls (luminance 62 cd m^–2^, background 0.7 cd m^–2^) was presented either on the right or the left side of the seal. These two balls were identical, their diameter is 7.7°, and their distance to the seal’s head 14° each. The alternative ball was presented on the other side of the seal after a stimulus onset asynchrony (SOA) of 8 ms up to 492 ms, i.e., 1–60 frames. Then, the participants were asked to hit the first presented ball on the tablet. There were 10 presentations for the practice, whose SOA varied from 328 to 115 ms. Then, in the experimental run, the SOA of the first presentation was 115 ms (14 frames), with two-down-one-up staircases randomly interleaved in linear steps of 33 ms (4 frames). A failsafe “bonus” trial (i.e., SOA of 492 ms) was interleaved to each of 7th to 13th trial. The experimental run was terminated after 30 trials.

#### Chinese Character Reading

A Chinese character reading task, which was utilized previously by Liu and McBride-Chang (2010), was used to examine the children’s reading ability at the word level. There were 100 simplified Chinese characters, listed in order of increasing difficulty level. Twenty characters were selected from Chinese language textbooks each of for Grades 2 to 6 (Shu et al., 2003). In this task, the participants were required to read 100 characters aloud one-by-one, and testing stopped when the child failed to recognize 15 consecutive characters. One point was awarded for each correct character; the maximum score for Chinese character reading was 100. Cronbach’s alpha was 0.97.

#### Control Measures

##### Raven’s standard progressive matrices

The children’s non-verbal intelligence was tested by Sets A and B of Raven’s Standard Progressive Matrices (Raven, 1996). For each test item, the children were asked to select an element from six options according to the pattern. Each correct was assigned one score, and the maximum score was 24. The Cronbach’s alpha was 0.78.

##### Morphological awareness

The compounding production task was used to test the participants’ morphological awareness (Liu et al., 2017). The participants were asked to listen to a scenario and were asked to use their imagination to create a novel word to represent the scenario properly. For example, one item in this task was “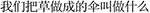 (What should we call an umbrella that is made of grass?” The answer for this item was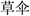 (grass umbrella). A five-point scale (0–4) was utilized to rate the answer of participants. There was a total of 31 items in this task, and the maximum score was 124. The Cronbach’s alpha was 0.86.

##### Orthographic knowledge

A Chinese character decision task (Li et al., 2012) was included to assess the participants’ awareness of orthographic patterns. There were four types of stimuli according to the radical and orthographic rules. The first type was “real” characters (i.e., 45 items) with correct radical and legal orthographic rules in Chinese (e.g., 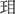). In addition, there were 15 pseudocharacters with correct radical but illegal orthographic rules (e.g., 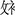), 15 items with incorrect radical but legal orthographic rules (e.g., 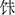), and 15 items with random combinations of strokes (e.g., 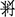). The children were instructed to select “real” characters from the 90 characters, although they had not learned any of these characters before. The 45 items with correct radical and legal orthographic rules (i.e., “real” character) should be judged as characters since they consist the correct radical and legal orthographic rules. In contrast, the other 45 characters should be judged as non-characters due to their incorrect components or illegal orthographic rules. One score was recorded if the child judged the pseudocharacter as a character or rejected others as a character. There was a total of 90 items, and the maximum score was 90. The Cronbach’s alpha was 0.81.

## Results

### Estimation of VTOTs

The visual thresholds were estimated for each run separately. The MATLAB toolbox psignifit (2.5.6^1^; cf. Wichmann and Hill, 2001) was utilized to analyse the SOAs with 75% correct responses. The thresholds were labelled as misses if (a) the threshold value and/or slope value was negative; (b) the threshold value was larger than 492 ms (i.e., SOA of a “bonus” trial); and (c) slope > 500 or slope > mean slope + 1 SD. In the final analysis, only the best threshold value (lowest SOA) of the two independent runs was included (Steinbrink et al., 2014). Accordingly, 79 VTOTs could be determined in 154 children. The correlations between all measures and the mean age and results of non-verbal intelligence, VWM, VTOTs, Chinese character reading, VSTM, morphological awareness, and orthographic knowledge are summarized in Table 1.

**TABLE 1:** Summary of correlations, means and standard deviations on age, non-verbal intelligence, visual working memory (VWM), visual temporal order threshold (VTOT), Chinese character reading, visual short-term memory (VSTM), morphological awareness, and orthographic knowledge.

	1	2	3	4	5	6	7	8	*M*	*SD*
1 Age	−								7.16	0.40
2 Non-verbal intelligence	–0.01	−	−	−	−	−	−	−	16.03	2.87
3 VWM	0.08	0.05	–0.15	−0.28*	0.06	–0.11	0.31**		2.94	1.37
4 VTOT	0.10	–0.05	0.30**	–0.08	0.24*	0.11			85.08	87.41
5 Chinese character reading	0.01	0.16	–0.07	–0.04	0.19				40.05	16.26
6 VSTM	–0.18	0.14	0.09	–0.09					3.09	1.58
7 Morphological awareness	0.01	0.05	0.24*						61.03	20.04
8 Orthographic knowledge	0.16	0.23*							57.47	9.25

*p < 0.05; **p < 0.01.

To investigate the effects of VTOTs and WM on Chinese character reading, we conducted a moderation effect analysis. Model Number 1 of the PROCESS macro for SPSS with 5,000 bootstrap samples was used, as suggested by Hayes (2017). The results are summarized in Table 2 and show significant predictive effects of VWM (*p* < 0.001), VTOTs (*p* = 0.02), and morphological awareness (*p* = 0.004) on Chinese character reading. Children with better performances in VWM, VTOTs, or morphological awareness had higher scores for Chinese character reading. More importantly, a significant interaction between VTOTs and VWM (*p* = 0.001) was found, which explained 11.45% of the variance in Chinese character recognition.

**TABLE 2:** The results of moderation effect analysis.

	*b*	*SE*	t	*p*	R^2^
VWM	2.66	1.26	2.11	0.04	0.46
VTOT	–0.04	0.02	–2.01	0.049	
VWM × VTOT	–0.06	0.02	–3.46	0.001	
Age	–2.96	4.80	–0.62	0.54	
Non-verbal intelligence	0.29	0.59	0.49	0.63	
VSTM	0.25	1.09	0.23	0.82	
Morphological awareness	3.52	1.18	2.98	0.004	
Orthographic knowledge	0.07	0.20	0.34	0.74	

*VWM, visual working memory; VTOT, visual temporal order threshold; VSTM, visual short-term memory.*

**FIGURE 2:**
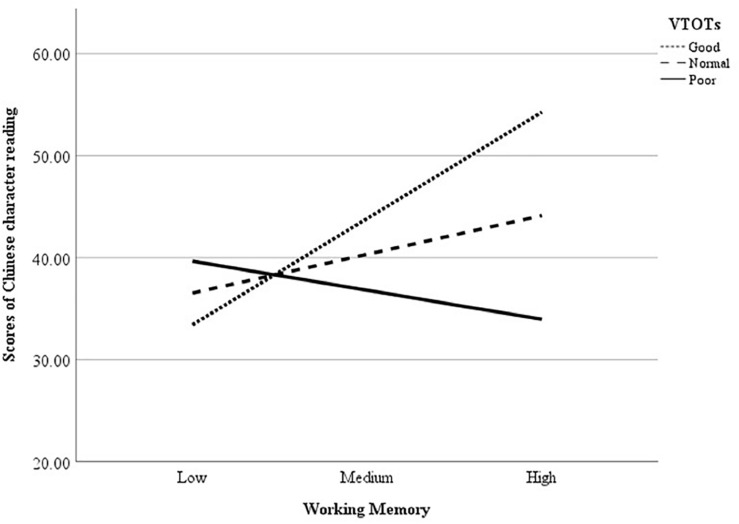
The relationships between working memory and Chinese character reading of children with different levels of VTOTs. VTOTs, visual temporal order thresholds.

To characterize the nature of this interaction further, the Johnson–Neymann (J–N) technique with 5,000 bootstrap samples was used, following suggestions and using the SPSS script provided by Hayes and Matthes (2009). The J–N technique allowed us to identify points directly in the range of the moderator variable (i.e., processing efficiency) where the effect of the predictor on the outcome transitions from being statistically significant to insignificant by finding the value of the moderator variable for which the ratio of the conditional effect to its standard error was equal to the critical *t* score. The conditional effect of VWM on Chinese character reading was (marginally) significant at a VTOT of 87.14, ß = 2.54, *SE* = 1.27, *t* = 2.00, *p* = 0.05, 95% CIs [0.00, 5.08], i.e., the 61th percentile of the distribution in our sample, as well as at a VTOT of 231.44, ß = −5.86, *SE* = 2.93, *t* = −2.00, *p* = 0.05, 95% CIs [−11.72, 0.00], i.e., the 22th percentile of the distribution in our sample. The relationship between WM and Chinese character reading was positively significant at VTOTs smaller than 87.14 ms, and negatively significant at VTOTs larger than 231.44 ms. This relationship was insignificant at VTOTs between these two thresholds (i.e., from 87.14 to 231.44) (see Figure 2).

## Discussion

The aim of the current study was to investigate the relationship between VWM and Chinese character recognition and the effect of processing efficiency on this relationship. Consistent with previous studies (Olson and Datta, 2002; Smith-Spark and Fisk, 2007; Chung et al., 2008; Peng et al., 2013; Wang and Yang, 2018; Wang et al., 2019), after controlling age, non-verbal intelligence, VSTM, morphological awareness, and orthographic awareness, the main effects of VWM and processing efficiency on Chinese character recognition were significant, which demonstrated their facilitating roles in Chinese children’s reading. Meanwhile, the current findings suggested a moderation effect of processing efficiency on the relationship between VWM and Chinese character recognition. To illustrate, among children with average or above processing efficiency, there was a positive association between VWM and Chinese character recognition. In contrast, for children with poor processing efficiency, the association became negative.

### VWM and Chinese Character Recognition

The results of the moderation effect analysis showed that the main effect of VWM on Chinese character reading was significant. The participants with better VWM capacity had higher scores for the Chinese character reading task, which supported a facilitating role of VWM capacity in Chinese character recognition. The VSTM did not predict the character recognition in the current sample, which suggested that the contribution of VWM to reading in Chinese should be due to the ability to process and maintain visual features simultaneously, rather than the ability to store information over a short period. An interpretation of these findings was that Chinese characters are constructed by visual components (e.g., strokes), which can be processed during the character recognition (Li et al., 2000; Liu et al., 2010). Keeping this visual information in the STM might be not enough for recognizing characters. As mentioned before, there are many similar characters that share visual components, such as 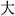
\da4\(big) and 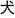
\tai4\(too). In contrast, VWM enables children to distinguish the target character from other similar ones in their mental lexicon while the processing of visual forms is running (Baddeley, 2003; Barrouillet et al., 2004).

Another possible reason for this relationship between VWM and Chinese character recognition might be related to the stroke order. The stroke order is required to achieve complete learning in Chinese (Tsai et al., 2012). The representation of stoke orders has been suggested as an independent representation for Chinese character reading (Shimomura, 1980; Flores d’Arcais, 1994). In addition to storing information, encoding and maintaining serial order information of perceived events is another important feature of WM (Ginsburg et al., 2017). With regard to the mechanism of the Corsi backward task for measuring children’s VWM in the current study (i.e., recalling the items in reverse sequence), in addition to remembering the items, the children were also required to remember and retrieve the visual sequences between items. Together, the reasons why VWM is beneficial to Chinese character acquisition and recognition may not only be that VWM can facilitate the storage of complex visual features of Chinese characters but also that it can facilitate the processing of serial information. In other words, VWM can help readers to process and maintain the forms and strokes of Chinese characters, and at the same time can assist them to integrate the strokes to construct the forms according to specific sequences.

### Processing Efficiency in VWM and Chinese Character Recognition

Consistent with previous studies performing the TOJ task in Chinese (e.g., Chung et al., 2008; Wang and Yang, 2018), in general, the present findings indicated that the children with higher visual processing efficiency (i.e., smaller VTOTs) had better performances on the Chinese character reading task. The positive link between visual temporal processing and reading in Chinese suggested that the ability of typically developing children to integrate visual information that converges in rapid succession is critical in orthographic processing in Chinese. Because of the opaque orthography of Chinese characters, character reading requires processing the elements and spatial configurations of a character. The literature has reported the importance of visual-orthographic processing in Chinese reading (McBride-Chang et al., 2005; Siok et al., 2009; Liu et al., 2015). Due to the aforementioned weak correspondence between orthography and phonology in Chinese, Chinese readers must recognize and integrate the orthographic segments of Chinese characters, and then further match the pronunciations stored in the mental lexicon with these Chinese characters. This process requires speed and relies heavily on visual processing at the same time, so it is reasonable to see its connection to visual temporal processing.

In addition, as mentioned, the literature reports that the specific sequence of strokes is a part of mental representations of Chinese characters (Giovanni, 1994; Parkinson et al., 2010). The current results imply a role for beginning readers’ sensitivity to determine the order of two rapidly presented non-literal visuals in reading (i.e., first graders). Consistently, it has been suggested that a visual-order processing deficit may be related to reading difficulty (e.g., Farmer and Klein, 1995; Booth et al., 2000). Together, these findings suggest that the rate of processing and retrieving visual features sequentially might influence orthographic coding, which could subsequently impact character recognition (Chung et al., 2008).

More importantly, a moderation effect of processing efficiency on the relationship between VWM and Chinese character recognition was revealed. Consistent with our assumption, the facilitating effect of VWM on Chinese character recognition was observed only in children with average or high processing efficiency. This finding supports that the correlation between VWM and Chinese character recognition should be due to an overlapping component between them, which is related to the efficient processing. According to the TBRS model (Barrouillet et al., 2007), this component should be the ability to switch between processing and maintenance. To illustrate, Chinese character recognition requires holding segments of orthography, but this information is subject to loss (due to either decay or interference). The TBRS model (Barrouillet et al., 2007) suggests that children with higher processing efficiency have more flexibility to allocate their cognitive resources to the stimuli in processing and maintenance. Thus, these children should perform better at permitting decoding, i.e., the ability to map written language onto speech with accuracy (Gough and Tunmer, 1986). The efficient processing could help the children to reach completion of the cognitive task, such as Chinese character recognition, before the requisite information is lost (Jensen, 1993; Miller and Vernon, 1996).

On the other hand, an inhibiting effect of VWM on Chinese character recognition was found in children with poor processing efficiency. One possible interpretation of the inhibiting effect might be that, as per our assumption, the children with low processing efficiency used more cognitive resources to process and maintain visual features of characters. Consequently, they did not have enough resources to activate the corresponding sounds, thus leading to a negative link between VWM and reading (Paas et al., 2003). In addition, the inhibiting effect might be related to the ability to inhibit distractors during processing. Specifically, as we mentioned before, many characters have similar orthography, so children with poor processing efficiency might have difficulty distinguishing them during character recognition. In other words, poor processing efficiency may elicit difficulties in locating their attention to target information and inhibiting noise during learning. Subsequently, their learning effect decreased (van der Sluis et al., 2007). For example, in Chinese, many characters can be components of other characters; for example, 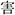
\hai4\(scare) can be a component of 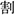
\ge1\(cut). However, their pronunciations are distinctive. There is a possibility that the information of the component 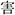
\hai4\(scare) is activated during the recognizing of the character 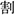
\ge1\ (cut).

In addition, the results of the participants from the 21th to 60th percentile of the distribution in our sample showed a non-significant correlation between VWM and Chinese character recognition. It should be noted that these participants’ processing efficiency was not defined as poor, but their performances still did not reach the average in the present sample. The non-significant correlation supports a possibility that, for these children with relative low processing efficiency, the processing and maintenance of visual features did not use too many cognitive resources to impede character recognition. However, the remaining cognitive resources did not produce any significant benefit to the following processing, such as activating sounds and inhibiting distractors in the mental lexicon.

On the other hand, these correlations between VWM and Chinese character recognition in children with different levels of processing efficiency might be due to learning of characters. It is possible that children with higher processing efficiency may have more efficient strategies for learning. The efficient strategies may both enhance their VWM capacity and character acquisition, which would further lead to a positive association between VWM and Chinese character recognition. There is evidence that Chinese readers do chunk strokes of a character into components, radicals, or even a whole to benefit memory. It has been reported that, at the early stage of learning to read Chinese, visual inputs of complex characters are decomposed into radicals when characters are being processed (Chua, 1999; Wu et al., 1999). In addition to improving the acquisition of characters, these visual chunking skills can save memory resources and improve VWM capacity (Anderson et al., 2013). To examine Chinese children’s character processing, one study utilized a delay-coding task in which children were shown a Chinese character briefly and were asked to reproduce it by writing it on paper with a pencil after the sample was removed (Pak et al., 2005). The results showed that children with more efficient approaches to chunking visual orthographic information had higher accuracies in the copying task, as well as reading ability, than those with less-developed visual chunking skills. Accordingly, it is possible that, with more efficient visual chunking strategies, such as chunking strokes as radicals, children’s VWM capacity and character learning are improved simultaneously. In turn, the positive correlation between VWM and character reading could be established. However, since we did not investigate the learning strategy directly in the present study, this interpretation should be explored in future studies.

### Limitations and Future Studies

First, students of only one grade were involved in the current study, and it has been reported that the relationship between VWM and reading may vary across grades (Gottardo et al., 1996; Berninger et al., 2010). With increasing grade levels, children’s reading experience accumulates, which may lead to automatic processing of characters. In other words, fewer cognitive resources are required for character reading. Thus, the relationships among processing efficiency, VWM, and Chinese character recognition might be different. To determine the role of grade in the relationships among these three variables, students of more grades should be involved in future studies. Additionally, a longitudinal study regarding the processing efficiency in VWM and Chinese character recognition should be conducted to help determine a more complete model of reading. Also, according to the criteria for VTOTs (Steinbrink et al., 2014), only 79 participants were taken into the moderation effect analysis. Although the J–N technique in PROCESS (Hayes, 2017) analyzed the data with 5,000 bootstrap samples to reduce the influence of the relative small sample size, a larger number of participants should be included in future studies to investigate whether the current findings would be the same.

Second, the current study focused on visual modality and orthographic processing in Chinese. Other modalities (e.g., auditory processing) and other aspects of characters (e.g., phonological processing) could be considered in the future. The findings regarding different modalities and character-related representations could provide a more complete understanding of the relationship between WM and reading. Third, only one reading ability was measured in the current study. Different reading tasks require different cognitive loads (Christopher et al., 2012; Oakhill and Cain, 2012), which may influence the relationship between VWM and reading and the moderation effect of processing efficiency. Other measures of reading at different levels, such as reading comprehension and reading fluency, should be considered in future studies. Finally, since (V)WM capacity and processing efficiency were measured in different tasks in the present study, the role of (V)WM efficiency in reading was not examined. Future studies should develop a task for measuring (V)WM capacity and efficiency simultaneously, which could improve the understanding of the mechanism of the relationship between WM and reading.

### Theoretical and Educational Implications

The present study found the facilitating effect of VWM on Chinese character recognition. In addition, the moderation effect of processing efficiency on the relationship between VWM and reading was revealed. Consistent with the view of TBRS model (Barrouillet et al., 2004), the current findings demonstrated that high processing efficiency could alleviate cognitive load and save memory space for further processing and, in turn, reading performance could be improved. On the other hand, to ensure accuracy, low processing efficiency may require more cognitive resources. This means that the cognitive load could be heavier for children with low processing efficiency, which may impair their subsequent processing.

According to the current findings, to improve children’s reading ability at the word level, strategies for enhancing children’s processing efficiency should be introduced in learning. Although WM capacity is limited, higher processing efficiency could allow more items to be held in memory for a longer time (Gough and Tunmer, 1986; Jensen, 1993; Miller and Vernon, 1996). In addition to presenting stokes and forms of characters, the regulations of Chinese characters, which can enhance the processing efficiency, should be highlighted in teaching. For example, for most Chinese compound characters, the phonetic radical, which is related to the pronunciation of characters, is always presented on the left side of a character, whereas the semantic radical, which is related to the meanings of characters, is always present on the other side. With this regulation, children can save cognitive resources required to remember the location of the phonetic and semantic radicals. They will not confuse a components’ location within a character, and then, they can use more cognitive resources for further processing to benefit their acquisition and recognition.

## Data Availability Statement

The datasets generated for this study are available on request to the corresponding author.

## Ethics Statement

The studies involving human participants were reviewed and approved by Human Research Ethics Committee, The Education University of Hong Kong. Written informed consent to participate in this study was provided by the participants legal guardian/next of kin.

## Author Contributions

ZX, L-CW, and DL designed and conducted tasks, analyzed the data, and co-wrote the manuscript. YC and LT conducted tasks, and contributed to the testing material design and preparation. All authors contributed to the article and approved the submitted version.

## Conflict of Interest

The authors declare that the research was conducted in the absence of any commercial or financial relationships that could be construed as a potential conflict of interest.

## Footnotes

1 http://bootstrap-software.org/psignifit
